# Single subject and group whole-brain fMRI mapping of male genital sensation at 7 Tesla

**DOI:** 10.1038/s41598-020-58966-9

**Published:** 2020-02-12

**Authors:** Sven P. R. Luijten, Ilse M. Groenendijk, Joan C. Holstege, Chris I. De Zeeuw, Wietske van der Zwaag, Bertil F. M. Blok

**Affiliations:** 1 Department of Urology, Erasmus Medical Center Rotterdam, Rotterdam, The Netherlands; 2 Department of Neuroscience, Erasmus Medical Center Rotterdam, Rotterdam, The Netherlands; 3 Spinoza Centre for Neuroimaging, Amsterdam, The Netherlands; 4 Netherlands Institute for Neuroscience, Amsterdam, The Netherlands

**Keywords:** Sensorimotor processing, Cortex, Urinary incontinence

## Abstract

Processing of genital sensations in the central nervous system of humans is still poorly understood. Current knowledge is mainly based on neuroimaging studies using electroencephalography (EEG), magneto-encephalography (MEG), and 1.5- or 3- Tesla (T) functional magnetic resonance imaging (fMRI), all of which suffer from limited spatial resolution and sensitivity, thereby relying on group analyses to reveal significant data. Here, we studied the impact of passive, yet non-arousing, tactile stimulation of the penile shaft using ultra-high field 7T fMRI. With this approach, penile stimulation evoked significant activations in distinct areas of the primary and secondary somatosensory cortices (S1 & S2), premotor cortex, insula, midcingulate gyrus, prefrontal cortex, thalamus and cerebellum, both at single subject and group level. Passive tactile stimulation of the feet, studied for control, also evoked significant activation in S1, S2, insula, thalamus and cerebellum, but predominantly, yet not exclusively, in areas that could be segregated from those associated with penile stimulation. Evaluation of the whole-brain activation patterns and connectivity analyses indicate that genital sensations following passive stimulation are, unlike those following feet stimulation, processed in both sensorimotor and affective regions.

## Introduction

Human sexual behaviour is characterized by inter- and intrapersonal intimacy. This includes tactile stimulation of the external genitalia, giving rise to both discriminative and affective sensations^1^. Despite the evident role of the brain in the perception of touch, it remains unclear how tactile input from the external genitalia is centrally processed in the healthy individual.

The primary and secondary somatosensory cortices (S1 and S2) are well-recognized as the main cortical areas involved in processing the discriminative properties of tactile input. Over the years, studies investigating genital touch in women and men have generally focused on S1 due to the counterintuitive location of the genitalia beneath the feet in the sensory homunculus^2,3^. Some confirm this location, recording cortical evoked responses deep in the medial wall of S1 during electrical stimulation of the dorsal nerve of the penis (DNP; principal sensory nerve of the penis)^4–6^. Later functional magnetic resonance imaging studies (fMRI) studies, however, demonstrated significant brain activation dorsolateral in the groin region of S1 in response to passive tactile stimulation of the external genitalia^7,8^. Findings regarding the involvement of S2 in processing tactile penile input show more consistency, demonstrating bilateral activation during both electrical^6^ and tactile stimulation^7,8^. Other brain regions have also been associated with processing the affective properties of touch. This includes not only the insula^9^, but also the anterior cingulate (ACC) and medial prefrontal cortex (mPFC) during tactile stimulation of the forearm^10^. Bilateral activation of the insula has been demonstrated in response to both electrical^6^ and tactile penile stimulation^8^. Activation of the midcingulate cortex (MCC), but not ACC, and mPFC have only been reported following tactile stimulation of the male genitalia^8^.

Recently, high field (7 Tesla) fMRI has emerged as a superior neuroimaging technique to study human brain function *in vivo*. Increases in signal-to-noise ratio (SNR) and blood-oxygenation-level dependent (BOLD) sensitivity at 7T^11,12^ have made it possible to acquire data with high spatial acuity and demonstrate robust activation in individuals. Recent studies showcasing these advantages include mapping the representation of individual digits^13^ and the lower limb^14^ in S1 at the single subject level. Given the substantial intersubject variability of neural representations as well as the need to treat patients as individuals, it has become eminently important to study human brain function at the level of individuals^13–15^. Moreover, signal advantages gained at 7 T can not only be observed at a limited field of view such as in S1, but also in more extended whole-brain acquisitions^16^ including the cerebellum^17^.

In the present study, we used 7T fMRI to acquire high-resolution neural representations of male genital sensation in the whole-brain at both single subject and group level. In order to do so, subjects underwent passive tactile stimulation of the genitalia in a way that minimized sexual arousal. Passive tactile stimulation of the medial aspect of the feet, performed in an equivalent manner as genital stimulation, served as a control task. The feet were deliberately chosen not only because of their location adjacent to the genitalia in the homunculus, but also as a more emotionally neutral stimulus. In addition, the medial aspect of the feet is involved in successful electrical therapies for an overactive bladder, like posterior tibial nerve stimulation^18^ or transcutaneous electrical nerve stimulation^19^. Therefore, current findings regarding supraspinal activation during tactile stimulation of the feet could provide more insight into which regions are targeted and affected by this treatment modality.

We hypothesized that the representation of the external genitalia is located in the groin region of S1, lateral to the feet. Furthermore, we hypothesized that passive tactile stimulation of the genitalia would lead to activation of brain regions associated with processing of both sensorimotor discriminatory (S1 & S2) and affective (insula, MCC and mPFC) properties of touch at both single subject and group level. In contrast, passive tactile stimulation of the feet would lead to activation of brain regions predominately associated with processing discriminatory properties of touch.

## Results

Four subjects were excluded from further analysis due to excessive spike head motion. The remaining thirteen subjects were included in first and second level of analyses. None of the subjects had an erection whilst undergoing tactile stimulation by the experimenter. Tactile stimulation of the penile shaft evoked significant activation superomedial and inferolateral in S1, S2, ventral premotor cortex (vPMC), posterior and anterior insula, posterior midcingulate gyrus (pMCG), mPFC, thalamus and cerebellum. Responses observed for left and right brushing were similar, and therefore added into a single contrast. Tactile stimulation of the feet evoked significant activation superomedial and inferolateral in S1, S2, vPMC, posterior insula, thalamus and the cerebellum.

### Representations in S1

**Figure 1:**
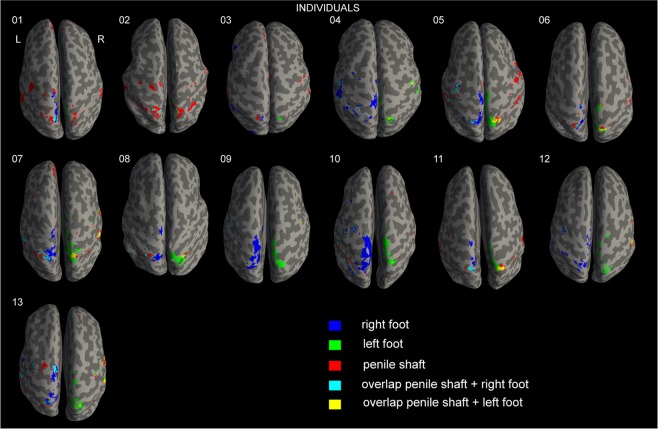
Single subject cortical activation patterns. Single subject activation maps (p < 0.05 FWE) from all individuals (n = 13) displayed on inflated anatomical images showing top-view. Legend in bottom-right corner indicating task specific color codes.

**Figure 2:**
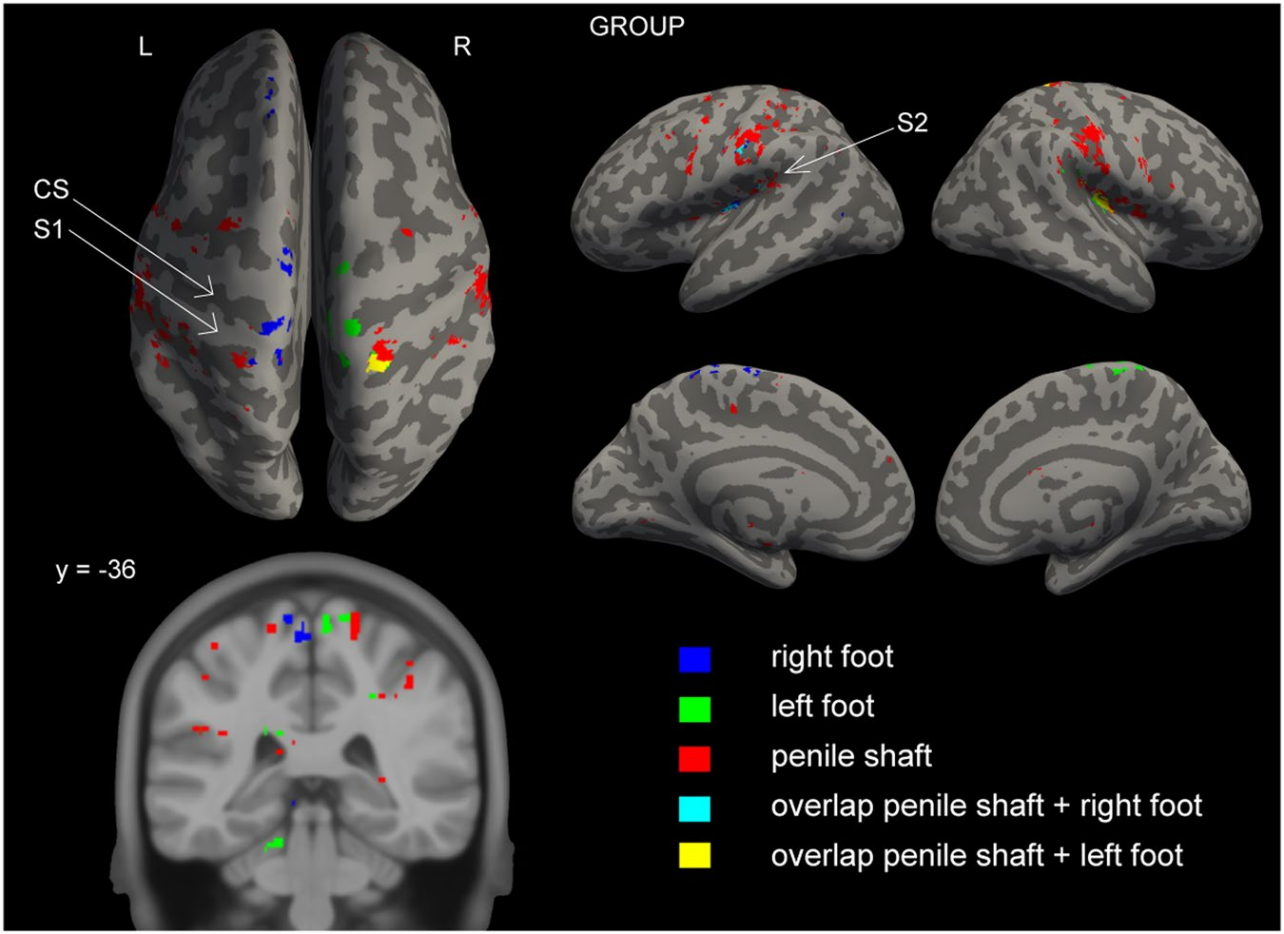
Group cortical activation patterns. Group activation maps (p < 0.005 uncorrected for multiple comparisons; n = 13) displayed on an inflated MNI template showing lateral, medial and top view. A coronal section from the MNI template (y = −36) is shown in the bottom-left corner, where the penile shaft is clearly located lateral to the feet in S1. Legend in the bottom-right corner indicating task specific color codes.

Tactile stimulation of the penile shaft and the feet evoked significant activation (p < 0.05 family wise error (FWE)) during whole-brain analysis in 11 out of 13 subjects (Fig. 1). Bilateral activation was observed superomedial and inferolateral in response to stimulation of the penile shaft. Unilateral activation was observed superomedial in the right hemisphere in response to stimulation of the left foot. Bilateral activation was observed superomedial in response to stimulation of the right foot. In addition, we also observed unilateral activation inferolateral in the left hemisphere. In single subjects, feet activation clusters extended anteromedial in S1 along the postcentral gyrus. Summation of these elongated clusters resulted in fractioning of feet activation clusters at group level (Fig. 2). Slight overlap was seen between penile shaft and feet activation clusters. Nevertheless, shaft clusters were consistently found lateral to the feet in the left and right hemispheres, both at single subject and group level.

### Whole-brain results

To further determine which cortical areas are implicated in processing tactile input from the genitalia and the feet outside of S1, we also examined whole-brain responses (Table 1). Tactile stimulation of the penile shaft elicited bilateral activations in S2, posterior and anterior insula, vPMC and the cerebellum. In single subjects, unilateral and bilateral activations were seen in the pMCG, mPFC and thalamus. At group level, unilateral activation in the pMCG was seen in the left hemisphere and bilateral activation was seen in the mPFC. We also observed subcortical activation in the medial and posterior regions of the thalamus, correlating with reported locations of the medial dorsal (MD) and ventral posterolateral (VPL) nuclei^20,21^. Tactile stimulation of the left foot elicited bilateral activations in S2, unilateral activation in the posterior insula in the right hemisphere, and unilateral activation in the cerebellum in the left hemisphere. Subcortical activation was observed posterior in the thalamus in the right hemisphere, presumably the VPL. Tactile stimulation of the right foot elicited bilateral activations in S2, posterior insula, and unilateral activation in the vPMC in the left hemisphere. Subcortical activation was observed posterior in the thalamus (VPL) in both hemispheres.

**Table 1:** Whole-brain group activation in response to stimulation of the penile shaft and feet versus rest.

Region	Hemisphere	Penile shaft				Foot (left)				Foot (right)			
		x	y	z	t-value	x	y	z	t-value	x	y	z	t-value
superomedial S1	L	−18	−38	68	3.39					−4	−38	72	7.90
	R	18	−40	68	3.77	6	−36	70	6.36	18	−40	68	6.07
S2	L	−42	−30	−22	4.13	−48	−36	26	4.36	−44	−30	20	5.39
	R	40	−30	24	6.19	46	−30	24	5.17	42	−30	22	5.26
inferolateral S1	L	−58	−16	34	6.57					−62	−16	36	5.63
	R	56	−18	32	4.38								
vPMC	L	−58	6	32	3.42								
	R	56	6	28	3.66								
posterior insula	L	−36	−16	16	3.13					−36	−18	16	6.74
	R	40	−12	14	4.33	34	−14	16	9.04	36	−16	16	5.93
anterior insula	L	−36	0	12	2.66								
	R	36	2	14	3.05								
pMCG	L	−12	−24	42	2.12								
	R												
mPFC	L	−4	52	26	1.89								
	R	12	60	28	1.81								
Thalamus	L	−12	−6	8	2.25					−22	−24	12	5.30
	R	18	−22	4	1.68	22	−22	12	4.70	16	−22	12	3.71
Cerebellum	L	−26	−54	−26	2.35	−14	−36	−22	5.09				
	R	20	−62	−24	2.73					20	−34	−26	4.11

Brain regions, MNI coordinates and peak t-values are listed. All activation for the penile shaft is reported using a global null conjunction analysis (p < 0.005 uncorrected for multiple comparisons, t-value > 1.52). All activation for the left and right feet are reported using a one sample t-test (p < 0.005 uncorrected for multiple comparisons, t-value > 3.05). S1: primary somatosensory cortex; S2: secondary somatosensory cortex; vPMC: ventral premotor cortex; pMCG: posterior midcingulate gyrus; mPFC: medial prefrontal cortex.

### Representations in the cerebellum

**Figure 3:**
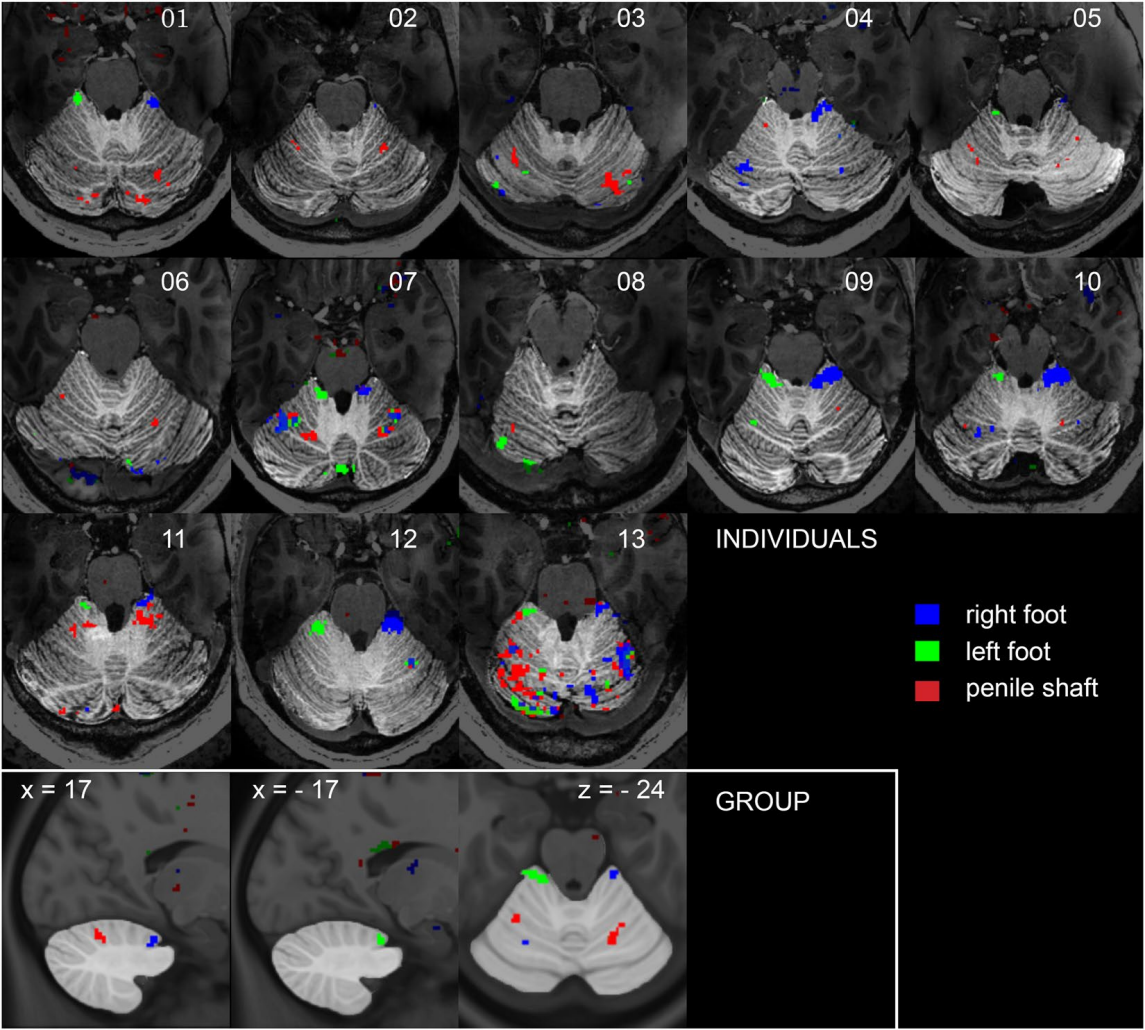
Single subject and group cerebellar activation patterns. Single subject activation maps from all individuals (p < 0.001 uncorrected for multiple comparisons) displayed on axial sections containing maximum number of representations. Group activation maps (p < 0.005 uncorrected for multiple comparisons, n = 13) displayed on sagittal and axial sections from the MNI template (x and z-coordinates in top-left corner indicate position in MNI space). Legend in bottom-right corner indicating task specific color codes.

At lower statistical thresholds, we observed significant activation in the anterior (lobules I-IV) and superior posterior lobe (lobule VI) of the cerebellum at both single subject (p < 0.001 uncorrected for multiple comparisons) and group level (p < 0.005 uncorrected for multiple comparisons) (Fig. 3). Shaft and feet representations were found in symmetrical locations in both cerebellar hemispheres across 8 out of 13 single subjects (Fig. 3). Tactile stimulation of the penile shaft evoked significant bilateral activation in lobule VI in the posterior lobe, part of the cerebrocerebellum. In 2 out of 13 individuals (#11, #13) activation was also seen just posterior to feet clusters in lobule IV. Tactile stimulation of the feet evoked significant unilateral activation in lobules IV in the anterior lobe, part of the spinocerebellum. In 4 out of 13 individuals (#04, 07, 12, 13) activation was also seen more posterior adjacent to the shaft clusters in lobule VI. At group level this was seen for the right foot in the contralateral cerebellar hemisphere (Fig. 3).

### Functional connectivity

**Figure 4:**
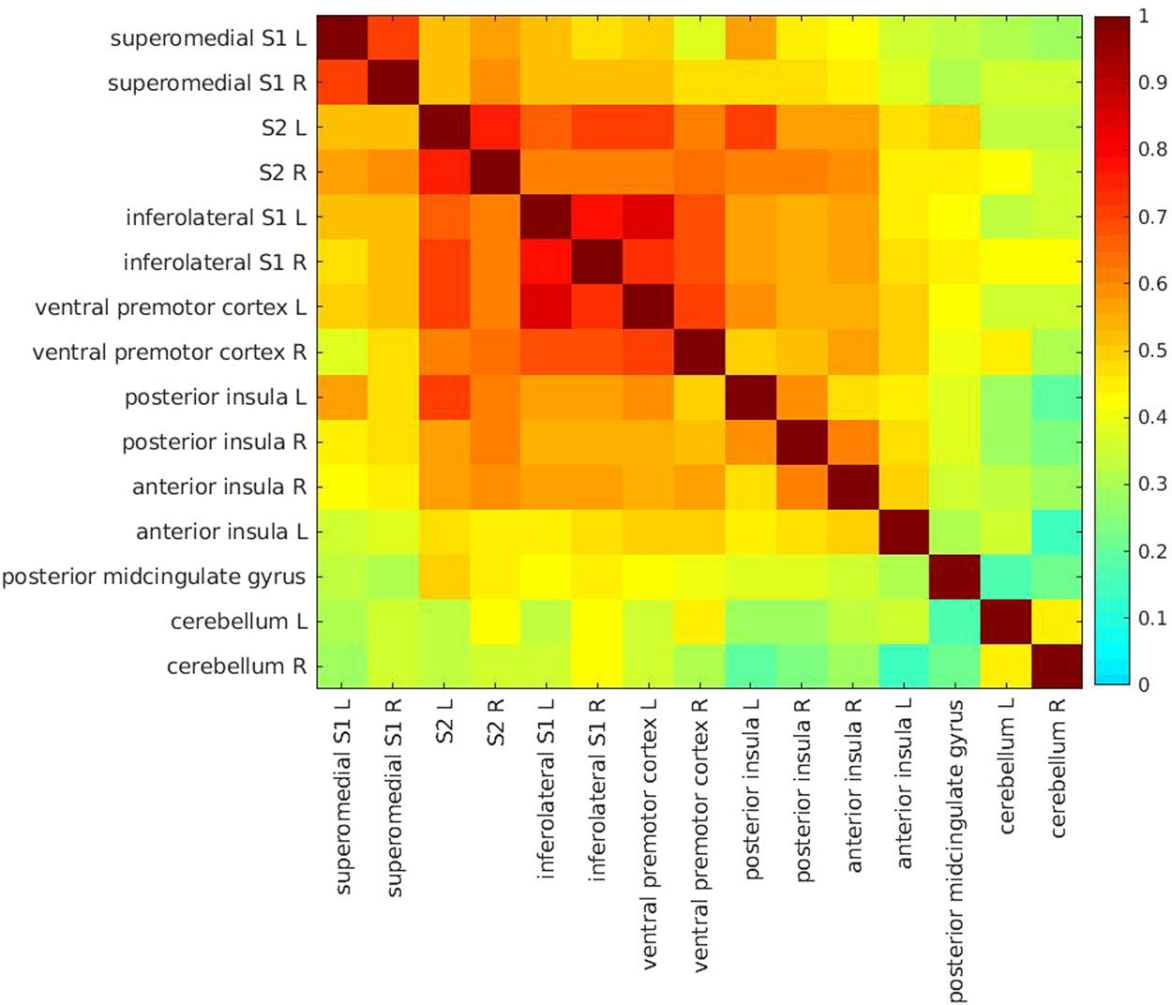
Mean connectivity matrix for penile shaft activation displaying connectivity between regions of interest. Individual connectivity matrices of 9 single subjects were first generated using a Pearson’s correlation coefficient, and subsequently used to generate a mean connectivity matrix. Regions of interest included are labeled along axes.

**Figure 5:**
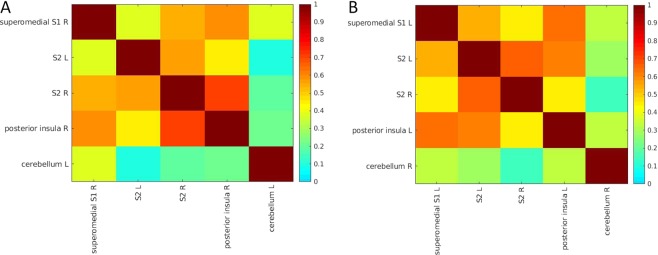
Mean connectivity matrix for left (**A**) and right (**B**) foot activation displaying connectivity between regions of interest. Individual connectivity matrices of 10 single subjects were first generated using a Pearson’s correlation coefficient, and used to generate a mean connectivity matrix. Regions included are labeled along the axes.

We also assessed the functional connectivity between timeseries of regions of interest (ROIs) for both functional tasks separately (Figs. 4 and 5). For the penile shaft, timeseries from the superomedial and inferolateral S1, S2, vPMC, posterior insula and the right anterior insula showed moderate correlation (range ρ 0.44–0.60). The left anterior insula showed weak correlation (range ρ 0.36–0.49) with the superomedial and inferolateral S1, S2, vPMC and posterior insula. Timeseries from the pMCG and cerebellum showed weak correlations overall with other ROIs.

For the left foot, timeseries from superomedial S1 in the right hemisphere showed a strong correlation with the S2 and posterior insula ROIs on the same side, whereas correlation with S2 in the left hemisphere was weaker (Fig. 5A). Timeseries from the cerebellum showed weak correlations overall with other ROIs. A similar, yet inverted, correlation pattern was observed for the right foot. Timeseries from superomedial S1 in left hemisphere showed high correlation with the S2 and posterior insula on the same side, whereas correlation with S2 in the right hemisphere was weaker (Fig. 5B). Timeseries from the cerebellum showed weak correlations overall with other ROIs.

### Distances

The distances between activation foci of penile shaft and feet representations in S1 and the cerebellum were measured (Table 2). In S1, vertex distances were measured over the cortical surfaces created during the inflation process in Freesurfer. This gives a more true representation of distances between cortical representations due to the high degree of folding of the postcentral gyrus. Since the cerebellum is not included in the inflation process, Euclidean distances were measured between cerebellar activation foci of penile shaft and feet representations. The distance between neighbouring body representations reflects the amount of cortical space taken up by those representations^22^. For example, a piano player will have larger digit representations than average, leading to a measurably larger distance between the thumb and the little finger. If, for example through underuse, the penile shaft representation gets smaller, this should have an effect on the distance between penile shaft and foot activation foci.

**Table 2:** Distances between group penile shaft and foot activation foci in S1 and cerebellum.

	Hemisphere	Distance (mm)
penile shaft - foot (S1)	L	27.5
	R	26.8
penile shaft - penile shaft (superomedial- inferolateral S1)	L	64.8
	R	63.1
penile shaft - foot (Cb)	L	20.9*
	R	28.1*

The distances between penile shaft and foot activation foci in both hemispheres at group level. Brain regions in which distances were measured are indicated in brackets. Distances measured in Euclidean space are indicated with an asterisk. S1: primary somatosensory cortex, Cb: cerebellum.

## Discussion

The present study is the first to investigate genital touch with the extensive field of view as supported by 7T imaging, and by doing so it provides novel data on the precise representations of the genitalia in the human brain, in particular those of hindbrain areas like the cerebellum that were often omitted with other approaches. By exploiting the increased BOLD sensitivity and specificity available at 7T, we obtained data with high spatial acuity and anatomical specificity. These clearly demonstrate that the genitalia are located in the groin region and not below the feet in S1. Furthermore, considerable differences were observed in whole-brain activation patterns in response to tactile stimulation of the genitalia as opposed to the feet. Tactile stimulation of the penile shaft evoked significant activations of discriminative (sensorimotor) and affective (emotional) brain regions, whereas tactile stimulation of the feet evoked significant activations of mainly discriminative brain regions. In addition, functional connectivity was assessed between activation clusters for both the genitalia and feet. This is the first study to report on functional connectivity of genital sensation.

Some have described the representation of the genitalia to be positioned in the medial wall below the representation of the feet in S1^4–6^, while others describe a more dorsolateral representation between the trunk and leg^7,8^. At both single subject and group level, our data clearly indicates that the genitalia are represented dorsolateral of the feet in S1 (Figs. 1 and 2), similar to what has been reported by previous studies using 3T fMRI^7,8^. Animal studies investigating genital representations have also described this location measuring extracellular recordings in primates^23^ and more recently using cortical microstimulation in rats^24^. Despite applying unilateral stimulation to the penile shaft, we observed bilateral activation in S1 irrespective of stimulation side. This corresponds well with findings showing that cutaneous regions situated in the midline of the body are represented bilaterally in S1^25^. Interestingly, studies using electrical stimulation of the DNP to locate the genitalia in S1 repeatedly demonstrated activation deep in the interhemispheric fissure. Cortical evoked responses elicited by electrical stimulation of the DNP were consistently located beneath those elicited by stimulation of the posterior tibial nerve^4–6^. It should be noted, however, that earlier techniques (e.g. EEG/MEG) used to measure brain responses offered poor spatial resolution. In addition, it is known that differences in evoked brain potentials can be observed when comparing electrical to tactile stimuli, further questioning this method when investigating the processing of physiological somatosensory stimuli^26^. In the present study, passive tactile stimulation of the medial aspect of the feet served as a control, analogous to electrical stimulation of the posterior tibial nerve. We expected to see activation in S1 lateralize in the contralateral hemisphere, as can be seen during stimulation of the left foot (Table 1). During stimulation of the right foot, however, significant yet weaker activation was also observed in the ipsilateral hemisphere (Table 1). Absence of lateralization in S1 has also been demonstrated during tactile stimulation of only the right and not the left hand in right-handed subjects^27^. The authors suggested this asymmetry is associated with hand preference and proficiency. Humans not only have a preference for left- or right-handedness, but also left- or right-footedness which can be seen in for instance football players^28^. In the present study we did not assess footedness prior to inclusion, however, we suggest ipsilateral activation in S1 during stimulation of the right foot may be the result of right-footedness.

Activation in S1 evoked during tactile stimulation of the feet extended anteromedial along the postcentral gyrus. Beneath this activation cluster, in most single subjects and at group level, activation was also observed deep in the interhemispheric fissure in response to tactile stimulation of the genitalia (Fig. 2; medial-view left hemisphere). At a closer examination, this area corresponds to the pMCG and not S1, which had been suggested previously^4^.

**Figure 6:**
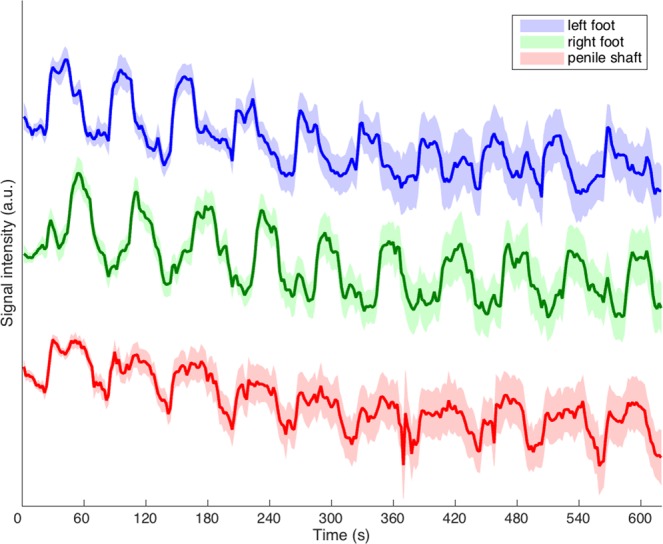
Mean signal intensity (in arbitrary units, a.u.) over time (in seconds, s) superomedial in S1 during tactile stimulation of the penile shaft and feet. The stimulation paradigm included 10 blocks (stimulation versus rest) each lasting 60 seconds with an additional rest block at the start resulting in a total scan duration of 620 seconds per task. For viewing purposes, curves were centered around separate baseline values. All timeseries were normalized prior to averaging across subjects. The shaded error bars indicate the standard error over subjects.

Although it was not the objective of this study, these data also allow a comparison of habituation effects during the functional tasks. Inspection of mean signal intensity curves superomedial in S1 show comparable habituation between shaft and feet stimulation (Fig. 6), indicating that task saliency was comparable.

Penile shaft and feet representations showed several areas of overlap (Fig. 1). Correspondingly, in women, overlap has been demonstrated between the genitalia and nipple^29^. This may have impeded earlier intraoperative mapping experiments by Penfield and colleagues, and it may partly clarify why genital sensations were so hard to induce^2^. Furthermore, this finding may also help to provide insight into why electrical therapies such as dorsal genital nerve^30^ and posterior tibial nerve^18,19^ stimulation share a similar inhibitory effect on bladder activity. Inferolateral in S1, we observed robust bilateral activation during tactile stimulation of the penile shaft and, to a lesser extent, in the left hemisphere during tactile stimulation of the right foot. Activation inferolateral in S1 has also been described during electrical stimulation of the clitoris^31^ and mechanical stimulation of the rectum suggesting this area may be involved in processing pelvic sensory information^32^. Furthermore, it has been argued that this cluster is in close proximity to the representation of the face in S1 and represents stimulus related activation rather than face/mouth movements due to discomfort^31^. We agree with this observation for the following reasoning. First, robust bilateral activation inferolateral in S1 was consistently seen in single subjects and the group. Prior to sensory tasks, subjects were instructed to lie still, breathe as they normally would and not make any movements. The current stimulus (brushing with a toothbrush) was well tolerated by participants and none reported discomfort after the scanning procedure. Hence, it is unlikely subjects consequently made similar mouth/face movements due to discomfort while they were explicitly instructed not to do so. Second, inferolateral activation clusters in S1 showed high functional connectivity to the superomedial S1 clusters and also other associative sensorimotor areas such as S2, the insula and vPMC (Fig. 4). This suggests that activation in this region is related to tactile genital stimulation and not due to co-occurring mouth/face movements.

Bilateral activation of the vPMC was observed during tactile stimulation of the penile shaft and unilateral activation of the vPMC was observed in the left hemisphere during tactile stimulation of the right foot. In both primates and humans, this area has been described to be sensitive to multisensory input, including tactile stimuli^33,34^. Accordingly, previous studies have demonstrated similar activation of this area during both tactile^8^ and electrical^31^ stimulation of the genitalia.

Activation of the posterior insula was observed during tactile stimulation of the penile shaft and the feet, whereas activation of the anterior insula was only observed during genital stimulation. Posterior insula activation has been described earlier and is associated with gentle touch processing^9,35^. Stimulation paradigms used in these studies included gentle stroking with a brush, similar to the stroking paradigm with a toothbrush in the present study. On the other hand, activation of the anterior insula was observed during stimulation of the penile shaft and not the feet. Other cortical areas solely activated during stimulation of the penile shaft include the pMCG and mPFC. These areas have been associated with the processing of affective/emotional properties of touch^10,36^, which fits well with the specific character of sensations (i.e. sexual or erotic) that may arise during tactile stimulation of the genitalia as opposed to the feet. In the current study, however, we did not assess potential sexual or erotic sensations experienced during stimulation making direct correlations not possible. Future research including psychometric measurements (e.g. by means of questionnaires) with both arousing and non-arousing stimuli is needed to determine whether activation of affective/emotional brain regions correlates with the perception of such sensations.

For the penile shaft, activation was observed posterior in the thalamus in the right hemisphere, corresponding to the VPL. In the left hemisphere activation as observed more anteriorly, corresponding to the MD (Table 1). Sensations of touch are processed through the dorsal column-medial lemniscus pathway projecting to the thalamus, in particular the VPL, and from thereon to the somatosensory cortex^20^. Our findings suggest the MD nucleus is also involved in processing genital touch. Accordingly, activation was also observed in the mPFC, where MD afferents project to^20^. In addition, electrophysiological studies in cats and rats have identified multiple bilateral subregions of the thalamus receiving inputs from the genital tract, including the VPL and MD^24,37^. Here, unilateral activation of the VPL in the left hemisphere and MD in the right hemisphere may, however, be the result of the small cluster size and weak BOLD signals measured in the thalamus using the current whole-brain acquisition protocol (Table 1). On the other hand, for the left foot, contralateral VPL activation was observed and for the right foot bilateral VPL activation was observed. Accordingly, contralateral S1 activation was observed for the left foot and bilateral S1 activation for the right foot.

Here we also mapped the representation of the penile shaft and feet in the anterior and posterior lobes of the cerebellum (Fig. 3). In line with previous findings, tactile stimulation of the feet evoked ipsilateral activation in lobule IV^38,39^. In some single subjects, activation was also observed more posterior in the cerebellum in both ipsilateral and contralateral hemispheres. At the group level, sparse activation was observed only in the contralateral hemisphere. We expected the genitalia would be represented just posterior to the feet in lobule IV, supporting previous fMRI studies in humans describing a somatotopical layout of the body in the anterior lobe of the cerebellum orientated anterior-posteriorly^38–40^. Surprisingly, in most single subjects and at the group level, genital representations were found more posterior in lobule VI in both cerebellar hemispheres (Fig. 3), seemingly posterior to the cerebellar hand representations^40^. In 2 individuals (#11, #13), activation was also observed adjacent to the feet representations in lobule VI, where we would have expected the genitalia to be represented. In these subjects, though, we also observed activation more posterior in lobule VI. When comparing cerebellar representations to those found in S1, the feet representations found anterior in lobule IV appear to be part of the somatomotor network^41^ also including the superomedial S1 feet and shaft representations. In contrast, the shaft representations found posteriorly in lobule VI may be part of a more associative frontoparietal network^41^ also incorporating the inferolateral S1 cluster.

In the present study, timeseries from cerebellar clusters showed low functional connectivity to any of the other ROIs. Regions that showed the highest degree of connectivity were the inferolateral S1 and vPMC in the right hemisphere. When inspecting the topographic organization of cerebrocerebellar circuits based on intrinsic functional connectivity, lobule VI is largely mapped to the inferolateral S1 and vPMC^41^. Our data suggest that these cerebellar representations of the genitalia in lobule VI belong to this particular cerebrocerebellar network. The relatively low functional connectivity demonstrated here may be the result of methodological differences such as task-based functional connectivity vs resting-state connectivity and sample sizes N = 9 vs N = 1000.

Functional connectivity was assessed for both functional tasks, no previous studies have reported on this. For the penile shaft, this was mainly done to see if we could demonstrate separate cerebral networks involved in processing genital touch (i.e. discriminative vs affective). Brain regions such as superomedial and inferolateral S1, S2, vPMC, the posterior insula and right anterior insula showed high functional connectivity to each other. On the other hand, the pMCG, left anterior insula and cerebellum showed low overall connectivity to other brain regions. Interestingly, for the feet, superomedial S1 representations showed high connectivity with S2 and the posterior insula on the ipsilateral side, whereas connectivity with S2 on the contralateral side was low. This suggests that, although bilateral activation of S2 was observed, there is some lateralization and dominance of the contralateral S2. Moreover, we observed high functional connectivity between the posterior insula and S2 in the same hemisphere, which is in accordance with the finding that these cortical areas are reciprocally connected^42^.

We acknowledge, however, that the task-based functional connectivity analysis in our study does not give a measure of intrinsic connectivity of neural networks in contrast to resting-state fMRI. In addition, measures of functional connectivity demonstrated here are related to our stimulation paradigm (i.e. brushing with a toothbrush). Other studies using different stimulation paradigms to investigate genital touch may produce different connectivity patterns.

The use of ultra-high field (7T) fMRI here provided considerable benefits compared to neuroimaging techniques used in previous studies investigating genital touch such as EEG, MEG and 3T fMRI^4–8^. Due to significant gains in SNR at 7T, we were able to acquire data with much higher spatial resolution (1.77 × 1.77 × 1.75 mm^3^), unmatched by previous studies. Furthermore, while no direct comparisons were made, it is plausible 7T fMRI offers increased BOLD sensitivity and allows detection of smaller effects facilitating increases in statistical strength when conducting both single subject and group analyses compared to fMRI at lower field strengths (1.5- & 3T)^17,43^. On the other hand, the relative contribution of physiological noise also increases at higher field strengths. 7T fMRI for instance, is more susceptible to false positive activation caused by subject head motion, potentially leading to higher exclusion rates in comparison to fMRI at lower field strengths. By employing a multiband EPI-sequence, whole-brain coverage including the cerebellum was achieved whilst preserving high spatiotemporal resolution. The current study is the first investigating genital sensation with such an extensive field-of-view and thereby the first to report on cerebellar representations of male genital sensation. This achievement may partly result from the fact that we placed a dielectric pad containing CaTiO_3_ posterior of subjects’ heads, which has been shown to increase both cerebellar T1-weighted anatomical coverage and detection of T2*-weighted BOLD signals^44^.

## Conclusion

In conclusion, using 7T fMRI, we present neural representations of genital sensation with unprecedented spatial resolution and whole-brain coverage in both single subjects and the group. We clearly show the genitalia are represented in the groin region in S1 and not below the feet. Whole-brain responses and additional connectivity analyses revealed that passive penile stimulation evoked significant activation in brain regions that can be segregated from those associated with feet stimulation. Genital sensations are processed in both sensorimotor and affective brain regions, whereas feet sensations are processed in sensorimotor regions. These differences may contribute to the specific character of sensations (i.e. sexual or erotic) that are associated with stimulation of the external genitalia.

## Materials and Methods

### Subjects

This study was conducted in agreement with the principles specified by the Declaration of Helsinki. Approval for the current study was given by the Medical Ethics Committee of the Erasmus Medical Center Rotterdam (METC 2015-451). All subjects provided written informed consent before entering the study. 17 healthy right-handed male subjects (mean age ± SD: 29.6 ± 7.8 years) participated in this study. Subjects were asked to take off their trousers and placed in a supine position on the MRI-bed.

### Stimuli and functional paradigm

All subjects completed the same scanning protocol, consisting of functional runs followed by a T1-weighted anatomical scan of the whole-brain for co-registration of functional data. Two sensory tasks were performed using a block paradigm. These tasks included subjects undergoing tactile stimulation of the penile shaft and medial aspect of the left and right foot. During both runs, an experimenter was positioned at the entrance of the scanner bore. Tactile stimulation was delivered using a commercially available toothbrush attached to a stick. The experimenter received audio cues indicating when and where to brush on MR-compatible headphones, generated in MATLAB using the Psychophysics Toolbox Version 3 (http://psychtoolbox.org/). The left and right penile shaft were brushed for a duration of 20 s respectively, followed by 20 s of rest (no brushing). This sequence was repeated 10 times with an additional rest period of 20 s at the start of both runs, resulting in a total scan time of 620 s per run. Brushing was done in a proximal to distal direction at a frequency of approximately 1 Hz and performed by the same experimenter for all subjects to minimize inter-subject stimulation variability^13^. For this study, a toothbrush was used to deliver tactile stimulation with the aim to mimic a physiological stimulus without inducing sexual arousal. The brushing of a toothbrush is a good alternative for human touch^7,45^ and can comfortably be executed while standing at the entrance of the scanner bore. Subjects were given a towel which they were instructed to place on the abdomen. Subsequently, subjects were instructed to place the penis on the towel in order to prevent skin-to-skin contact with the thigh and abdomen during the tactile stimulation. Prior to the actual scanning session, all subjects underwent a training session in a mock scanner. This gave subjects the opportunity to get acquainted with the tactile stimulus (brushing of a toothbrush). During this training session, tactile stimulation was delivered in a similar manner as described above.

### Data acquisition

All functional and structural data were acquired on a 7T MRI scanner (Philips Achieva) using a volume transmit coil and a 32-channel receive coil (Nova Medical). Functional data was acquired using a multiband echo planar imaging (mb-EPI) sequence with multiband factor 2. Whole-brain coverage, including the anterior lobe of the cerebellum, was achieved using the following parameters: voxel size 1.77 × 1.77 × 1.75 mm^3^; matrix size: 104 × 127; FOV = 184 × 223 mm^2^; number of slices: 70; TR/TE = 2000/25 ms; flip angle = 70°; in-plane SENSE factor R = 3. Whole-brain anatomical data was acquired using the MPRAGE sequence with the following parameters: voxel size 0.7 × 0.7 × 0.7 mm^3^, matrix size: 352 × 353, FOV = 246 mm; number of slices: 249; TR/TE = 4.4/1.97 s, SENSE factors R = 1.6 (anterior-posterior) and R = 1.5 (right-left); total acquisition time 8’35”. In addition, to account for signal loss in infratentorial areas, a dielectric pad containing calcium titanate (CaTiO_3_)^46^ was placed posterior of the subjects’ heads^46^.

### Image preprocessing

All data was reconstructed on an offline workstation using dedicated reconstruction software (ReconFrame, Gyrotools, Zürich, Switzerland). Further data processing was done in SPM12 (Wellcome Trust Center for Neuroimaging, London, UK). Pre-processing steps included joint image realignment of all four functional runs, co-registration of the anatomical image to the resulting mean functional image and smoothing of functional data with a Gaussian kernel (FWHM 2.5 mm). For the extraction of peak activation coordinates, functional data was normalized to the standardized brain template of the Montreal Neurological Institute (MNI). Additionally, inflated cortical surfaces were created in Freesurfer (http://surfer.nmr.harvard.edu/) using single subject anatomical images and the MNI template. In order to aid the inflation process, all images were first bias corrected (bias FWHM = 18, sampling distance = 2) and resliced to 1 mm isotropic in SPM.

### Whole-brain analyses

First level statistical analysis was conducted using the General Linear Model (GLM). Each functional task was modeled as a boxcar convolved with a canonical hemodynamic response function (HRF) and temporal derivative as basis functions. Realignment parameters were added as nuisance regressors to account for confounding motion effects. The response for each task was estimated independently from the others. Activation maps for tactile stimulation of the left and right penile shaft showed a high degree of overlap in both hemispheres, and were therefore conjoined into a single contrast using a global null conjunction analysis^47^ thresholded at p < 0.05 voxel-based FWE). Activation maps for tactile stimulation of the left and right foot were generated as separate contrasts and thresholded at p < 0.05 FWE.

Second level statistical analysis was conducted using a one-sample t-test on individuals’ task responses. Likewise, at group level, left and right shaft contrasts were conjoined using a global null conjunction analysis p < 0.005 uncorrected for multiple comparisons. Activation maps for tactile stimulation of the left and right foot were again thresholded at p < 0.005 uncorrected for multiple comparisons. Both single subject and group level cortical activation maps were projected on inflated cortical surfaces created in Freesurfer and sampled halfway the mid-cortical depth in order to avoid vascular artifacts at the pial surface.

### Functional connectivity analyses

To further evaluate activation of different networks (i.e. discriminative vs affective), we computed the correlation between timeseries from different ROIs in single subjects. Timeseries were extracted from individual pre-processed contrast images, which were realigned, co-registered and smoothed as described earlier. For the penile shaft, ROIs included were S1, S2, vPMC, posterior and anterior insula, pMCG and the cerebellum. ROIs were isolated using individuals’ contrast images and successfully identified in 9 out of 13 individuals. Activation in the thalamus and mPFC could only be observed in 4 and 5 subjects respectively, and were therefore not included in the functional connectivity analysis. For the feet, included ROIs were the S1, S2, posterior insula and cerebellum. Again, ROIs were isolated using individuals’ contrast images and were successfully isolated in 10 out of 13 individuals. Overlapping activation clusters were manually separated in ITK-SNAP (http://www.itksnap.org/). Subsequently, voxel timeseries were extracted from each ROI per single subject and denoised for signal arising from white matter, gray matter and cerebrospinal fluid using linear regression. Connectivity was defined as the linear correlation between timeseries of different ROIs, which was computed with the Pearson’s correlation coefficient. Single subject correlation matrices were used to compute a mean correlation matrix for both the penile shaft and feet.

## Data Availability

The datasets analyzed during the current study are available in NIFTI format for interested researchers. Please contact the corresponding author to make a request.
